# Supplementary material for: Chromothripsis during telomere crisis is independent of NHEJ, and consistent with a replicative origin
Source: Genome Res. 2019 May;29(5):737–49. doi: 10.1101/gr.240705.118 (PMC6499312; doi:10.1101/gr.240705.118)
Supplement: Supplemental Material [file supp_gr.240705.118_Supplemental_file_1.zip › contigs/annotated_contigs/DB83/contig.2.DB83_length_775_mean_cov_4.45161290323.docx]

**DB83_length_775_mean_cov_4.45161290323**

```
GTGAGAGATGAGTCTAACCTGTAAAGCTGGATGAGACATTCAAGATTAATGTTACCATATGAGAACCCTTTTTAACACAATCTAACCTT
 >chr3:157922446-157922804 - E=2e-197 p=1e-03
TCTGTTTTATTCACTCTCTCATATTTCTTACCTTTAAAATTCCCCATATAATGTTTTATATAATATAAATTTTATGCAATTACACAAGT

ATTATTCTAAAAAAGTAAACTTTGGTGAGGTGGAAGGCAAAAATTTAATGACCAGTAAAAATCATAGCTTTCTTCCACAGCCATGTAAT

TTTTAAAAACAAAACCAAAAGAAGAAAAAATTTTAAAAGGAACCATAAGCAAGGCAGTTCCTTGATTGACAAGTTTTATTCATTAAGCA

TA|AAATAATATGCTTAAT|GACATAAAATAATCATAAAATGATAGACTTAAATCTAATTATATCAATAAGTACATTAGATATTAATAA
                   >chr3:157917931-157918332 - E=2e-226
ACAAAATATTGTAAAAAGGCAGTTTGTCAAATTAATAAAAAGCAATACTGTATGCCATCTAAAACAACAACTACATGCTATCTACAAAA

TATGTACTTTAAATAAGCAGACACAGATAGGTTAAAAGTAAATGGCCAGGCCGGGCGTGGTAGCTCACGCCTCTAATCCCAGCACTTTG

GGAGGCTGAGGTGAGGGGATCACGAGGTCAGGAGATCGAGACCATCCGGGCTAACATGGTGAAACCCTGTCTCTACTAAAAAATACAAA

AAATTAGCTGGGCATGGTGGCGGGTGCCTGTAGTCCCAGCTACTTGGGAGGCTGAGGCAGAAGAA
```
